# Tocotrienols exhibit superior ferroptosis inhibition over tocopherols

**DOI:** 10.1038/s41598-025-34673-1

**Published:** 2026-01-07

**Authors:** Hao Yang, Junya Ito, Taiki Maejima, Shinnosuke Kimura, Hikaru Ino, Yusuke Hirata, Atsushi Matsuzawa, Sho Kobayashi, Eikan Mishima, Kiyotaka Nakagawa

**Affiliations:** 1 Laboratory of Food Function Analysis, Graduate School of Agricultural Science, Tohoku University, Sendai, Miyagi 980-8572 Japan; 2 Division of Food and Redox Biology, Education and Research Center for Food Agricultural Immunology, Graduate School of Agricultural Science, Tohoku University, Sendai, 980-8572 Japan; 3 Institute of Metabolism and Cell Death, Molecular Targets & Therapeutics Center, Helmholtz Munich, Neuherberg, 85764 Munich, Bavaria Germany; 4 Laboratory of Health Chemistry, Graduate School of Pharmaceutical Sciences, Tohoku University, Sendai, Miyagi 980-8578 Japan; 5 Department of Food, Life and Environmental Science, Faculty of Agriculture, Yamagata University, Tsuruoka, Yamagata 997-8555 Japan; 6 Department of Redox Molecular Medicine, Tohoku University Graduate School of Medicine, Sendai, Miyagi 980-0875 Japan

**Keywords:** Vitamin E, Lipid peroxidation, Antioxidant, Regulated cell death, Biochemistry, Cell biology, Drug discovery

## Abstract

Ferroptosis is a regulated form of cell death characterized by iron-dependent phospholipid peroxidation. Micronutrients play a critical role in modulating cellular sensitivity to ferroptosis, with vitamin E being a well-known ferroptosis inhibitor through its lipophilic antioxidant property. While vitamin E naturally exists in two forms of tocochromanols, namely tocopherols and tocotrienols, each comprising α, β, γ, and δ analogs with distinct antioxidant capacities and metabolic profiles, only α-tocopherol has been the focus of most ferroptosis studies. Here, we compared the efficacy of all vitamin E analogs, including tocopherols, tocotrienols and Trolox, in terms of their ability to prevent ferroptosis and their antioxidant activities linked to suppressing lipid peroxidation. We demonstrate that tocotrienols exhibit superior ferroptosis inhibition over tocopherols. Tocotrienols prevented ferroptosis induced by both pharmacological models (inhibition of GPX4, xCT or glutathione synthesis) and a genetic model of inducible *Gpx4* knockout at significantly lower concentrations than tocopherols (e.g., EC_50_ of 0.12 μM for α-tocotrienol and 2.0 μM for α-tocopherol in *Gpx4* knockout cells). Consistent with this, tocotrienols exhibited greater antioxidant activities in suppressing lipid peroxidation. These findings highlight the distinct properties of vitamin E analogs in regulating ferroptosis and underscore the superior potential of tocotrienols as potent ferroptosis suppressors.

**Supplementary Information:**

The online version contains supplementary material available at 10.1038/s41598-025-34673-1.

## Introduction

Ferroptosis is a form of regulated cell death characterized by iron-mediated lipid peroxidation triggered by the disruption of cellular redox homeostasis^1^. This cell death modality has gained great attention because of its link with a variety of pathological conditions, such as neurodegenerative diseases and acute organ injuries^2,3^. The regulation of ferroptosis has, therefore, emerged as an important target of research for potential therapeutic intervention.

**Fig. 1:**
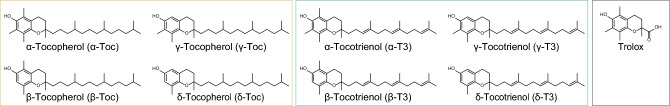
Structure of vitamin E analogs. Chemical structures of tocopherols, tocotrienols and Trolox are shown.

Cells employ various systems to suppress excessive lipid peroxidation to prevent ferroptosis^4,5^. For example, the cysteine/glutathione (GSH)/glutathione peroxidase 4 (GPX4) axis provides the prime defense against ferroptosis by converting potentially detrimental lipid hydroperoxides^6,7^. In addition, the scavenging of lipid radicals by antioxidants, such as the reduced forms of coenzyme Q_10_ and vitamin K, and tetrahydrobiopterin, plays a key role in preventing ferroptosis^8–11^. Among them, vitamin E has been recognized as an important endogenous ferroptosis suppressor, as nature’s premier antioxidant, which can scavenge lipid radicals and prevent lipid peroxidation^12^. While vitamin E comprises eight naturally occurring analogs, namely the α-, β-, γ-, and δ-analogs of both tocopherols and tocotrienols (Fig. 1), each exhibiting distinct antioxidant capacities and metabolic profiles^13–15^, only α-tocopherol has been the primary focus in ferroptosis-related research, as it is the most common form of vitamin E present in human blood and tissues.

Tocotrienols, which share a similar structure to tocopherols but differ in possessing three double bonds in their isoprenoid side chain, have been found to exhibit unique biological activities that are not shared with tocopherols^16–18^. Previous studies have reported that, compared with tocopherols, tocotrienols exhibit stronger cytoprotective effect against oxidative stress^19–21^ and higher antioxidant potency against lipid peroxidation in rat liver microsomal membranes^22^. In contrast, the antioxidant potency of tocopherols and tocotrienols appears to be comparable when assessed using assays such as the 2,2-diphenyl-1-picrylhydrazyl (DPPH) assay^15,23^, a conventional method for evaluating the antioxidant activity of compounds^24^. However, it is well recognized that antioxidant capacity measured by DPPH assay does not necessarily correlate with ferroptosis-preventing effects of the compounds^25^. Moreover, although the antiferroptotic effect of α-tocopherol has been extensively studied, direct comparisons between tocotrienols and tocopherols in the context of ferroptosis inhibition remain limited. In this study, we aimed to compare the ferroptosis-suppressing effects of vitamin E analogs, revealing the superior ferroptosis-inhibiting potency of tocotrienols over tocopherols.

## Results

### Tocotrienols possess stronger antiferroptotic activity than tocopherols

**Fig. 2:**
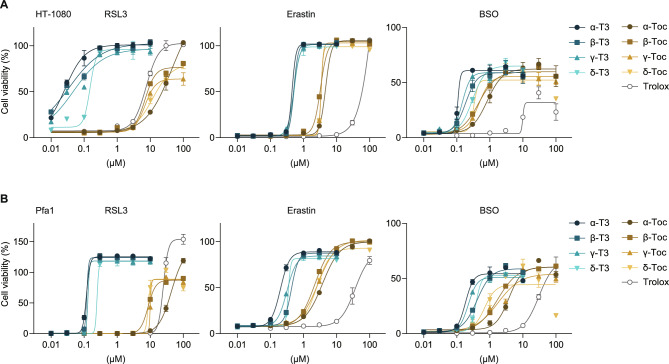
Ferroptosis-preventing activity of vitamin E analogs in HT-1080 and Pfa1 cells against RSL3, erastin and BSO. (**A and B**) Cell viability of HT-1080 (in A) and Pfa1 cells (in B) pretreated with tocotrienols (T3, 0–10 µM), tocopherols (Toc, 0–100 µM), or Trolox (0–100 µM) and treated with ferroptosis inducers: RSL3 (0.5 µM for 24 h), erastin (1 µM for 48 h), or BSO (100 µM for 72 h). Data are presented as mean ± SD (n = 3).

We used high-purity (> 99%) reagents of nine vitamin E analogs, including tocopherols, tocotrienols, and Trolox (Fig. 1). We assessed the ability of the vitamin E analogs to prevent ferroptosis in human sarcoma HT-1080 cells, a cell line frequently used in ferroptosis research because of their high susceptibility to ferroptosis^26^. Ferroptosis is pharmacologically induced by well-established ferroptosis inhibitors: (*1S,3R*)-RSL3 (RSL3; a GPX4 inhibitor)^7,27^, erastin (an inhibitor of the cystine-glutamate antiporter)^1^ and *L-*buthionine sulfoximine (BSO; a GSH synthesis inhibitor)^28^ (Supplementary Fig. 1A and B). As a result, while all vitamin E analogs prevented RSL3-induced cell death, the effective concentration required for protection differed between tocopherols and tocotrienols. Tocotrienols inhibited RSL3-induced ferroptosis at significantly lower concentrations than tocopherols did (Fig. 2A and Supplementary Fig. 2A). For instance, tocotrienols completely prevented ferroptosis at concentrations below 1 μM, whereas tocopherols required concentrations above 10 μM to inhibit RSL3-induced ferroptosis in both cells. Tocotrienols also effectively prevented ferroptosis induced by erastin and BSO in lesser amounts than tocopherols (Fig. 2A and Supplementary Fig. 2A). The ferroptosis-preventing ability of the vitamin E analogs was also evaluated using a lactate dehydrogenase (LDH) release assay, a marker of cell death associated with plasma membrane rupture. Tocotrienols markedly reduced LDH release induced by erastin and BSO at much lower concentrations than tocopherols (Supplementary Fig. 2B).

Furthermore, we assessed the ferroptosis-preventing ability of the vitamin E analogs in non-cancer cells, using mouse embryonic fibroblasts Pfa1^29^. The superior ferroptosis prevention effect of tocotrienols was consistently observed in Pfa1 cells treated with RSL3, erastin and BSO (Fig. 2B). These results suggested that tocotrienols possess stronger antiferroptotic activity than tocopherols across multiple ferroptosis triggers.

### Tocotrienols effectively inhibit ferroptosis induced by Gpx4 deletion

**Fig. 3:**
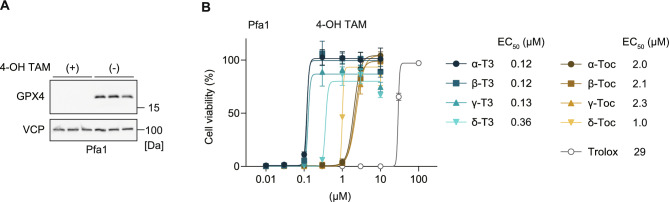
Ferroptosis-preventing activity of vitamin E analogs in Pfa1 cells genetically deleted *Gpx4*. (**A**) Immunoblot analysis of Pfa1 cells treated with or without 4-OH-TAM (1 µM, 72 h) in the presence of liproxstatin-1 (0.5 μM). VCP was used as a loading control. (**B**) Cell viability of Pfa1 cells treated with 4-OH-TAM (1 µM) and co-treated with tocotrienols (0–10 µM), tocopherols (0–100 µM), or Trolox (0–100 µM) for 72 h. Data are presented as mean ± SD (n = 3).

Given that ferroptosis triggered by different mechanisms can result in distinct phenotypes, potentially due to off-target effects of ferroptosis-inducing compounds or epiphenomena occurring alongside ferroptosis^5^, we also evaluated the ferroptosis prevention effect of vitamin E on ferroptosis induced via a genetic approach. Pfa1 cells are widely used in ferroptosis research, where treatment with 4-hydroxytamoxifen (4-OH TAM) induces *Gpx4* deletion and ferroptosis^29^. To confirm the validity of this genetic model, we verified that 4-OH TAM efficiently depleted GPX4 expression by immunoblotting in Pfa1 cell (Fig. 3A). in this model, tocotrienols prevent ferroptosis by inducible *Gpx4* deletion in lesser amounts than tocopherols (Fig. 3B). The EC_50_ was 0.12, 0.12, 0.13 and 0.36 μM in α, β, γ, and δ-tocotrienol; 2.0, 2.1, 2.3 and 1.0 μM in α, β, γ, and δ-tocopherol, whereas Trolox showed markedly lower potency (EC_50_ 29 μM). Thus, across both chemical and genetic ferroptosis models, tocotrienols reproducibly exhibited superior antiferroptotic activity.

### Antioxidant activity of vitamin E analogs assessed by the FENIX assay and BODIPY 581/591 C11 assay

**Fig. 4:**
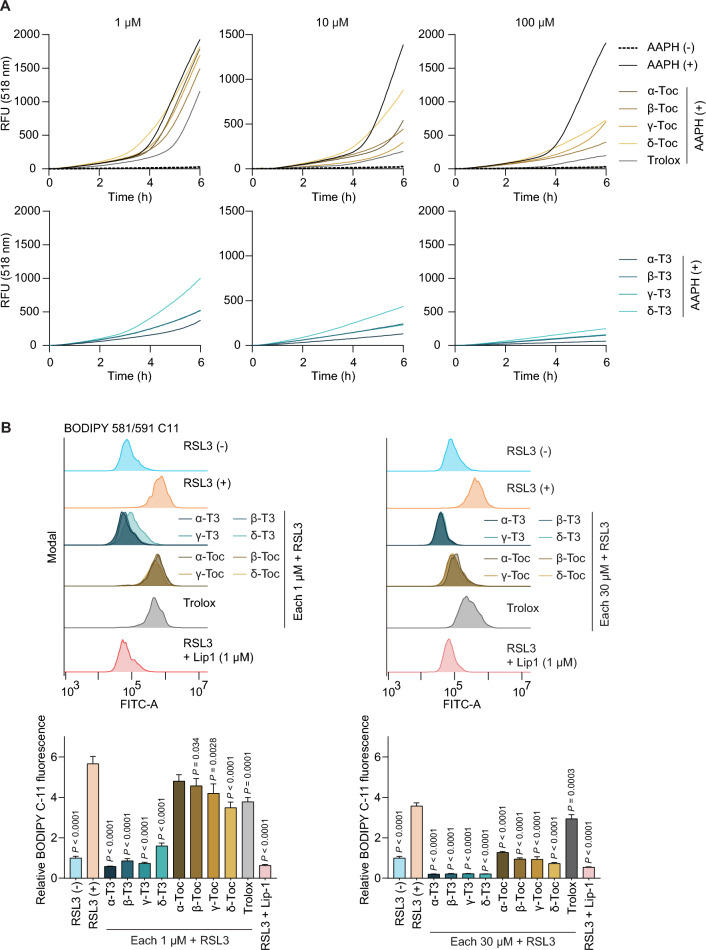
Antioxidant activities of vitamin E analogs evaluated by the FENIX assay and BODIPY 581/591 C11 assay. (**A**) The antioxidant activities of vitamin E analogs (1, 10 and 100 μM) were evaluated using the FENIX assay. Fluorescence intensity, corresponding to the oxidation level of STY-BODIPY, was measured. A negative control without AAPH, a radical initiator, was included. RFU, Relative fluorescence units. (**B**) BODIPY 581/591 C11 lipid peroxidation assay. (Upper) After 1 h of preincubation with vitamin E (1 and 30 μM) or Lip-1 (1 μM), HT-1080 cells were treated with RSL3 (0.5 μM, 2 h) and subjected to flow-cytometric analysis of oxidized BODIPY 581/591 C11 fluorescence. (Lower) Fluorescence values were normalized to the RSL3(–) condition (set to 1.0). Statistical analysis was performed by one-way ANOVA using the RSL3( +) group as the reference, followed by Dunnett’s post hoc test. Values of *P* < 0.05 are indicated.

Next, the antioxidant activity of each vitamin E analog against lipid peroxidation was assessed using a fluorescence-enabled inhibited autoxidation (FENIX) assay^25^, in which radical-initiated liposomal lipid peroxidation is monitored fluorometrically through the competitive oxidation of a fluorescent probe, STY-BODIPY. Antioxidant activity evaluated by this assay has been reported to correlate well with a compound’s antiferroptotic potential^25^. The effect of each vitamin E analog was evaluated at concentrations of 1, 10 and 100 μM (Fig. 4A and Supplementary Fig. 3). As a result, tocotrienols efficiently suppressed lipid peroxidation than tocopherols under cell-free liposomal conditions, with α-tocotrienol exhibiting the highest suppressing effect among the tocotrienols. This result supports the notion that tocotrienols exhibit higher radical-trapping efficiency within lipid bilayers.

We additionally examined lipid peroxidation at the cellular level using the fluorescent lipid peroxidation probe BODIPY 581/591 C11 (Fig. 4B). RSL3 treatment induced a robust increase in oxidized BODIPY 581/591 C11 in HT-1080 cells. Pretreatment with tocotrienols markedly reduced lipid oxidation indicated by oxidized BODIPY 581/591 C11, whereas tocopherols required higher concentrations to achieve similar effects (Fig. 4B). These data demonstrate that tocotrienols more effectively suppress intracellular lipid peroxidation, providing a mechanistic basis for their stronger antiferroptotic activity.

### Evaluation of the cellular toxicity of vitamin E analogs

**Fig. 5:**
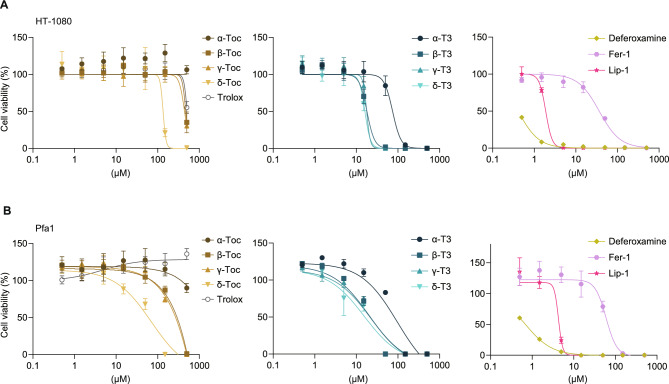
Evaluation of the cellular toxicity of vitamin E analogs. Cell viability of HT-1080 and Pfa1 cells treated with vitamin E analogs or ferroptosis inhibitors, including ferrostatin-1 (Fer-1) and liproxstatin-1 (Lip-1) and deferoxamine for 72 h. Data are presented as mean ± SD (n = 3).

We additionally evaluated the cellular toxicity of each vitamin E analog in HT-1080 and Pfa1 cells. After treatment with each vitamin E analog as well as with well-established ferroptosis inhibitors ferrostatin-1 (Fer-1)^1,30^, liproxstatin-1 (Lip-1)^6^ and deferoxamine^1^ for 72 h, the cell viability was assessed. As a result, tocotrienols exhibited cytotoxicity at lower concentrations than tocopherols, but were less cytotoxic than Lip-1 and deferoxamine, and showed toxicity comparable to Fer-1 (Fig. 5A and B).

## Discussion

**Fig. 6:**
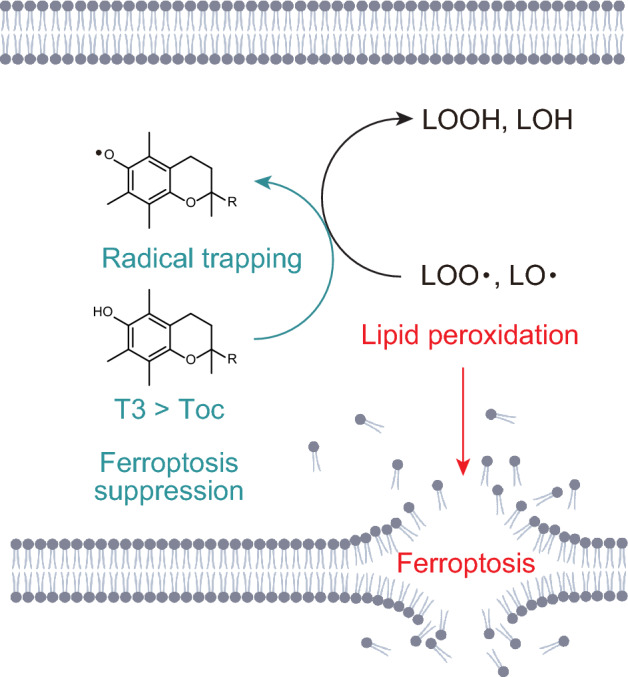
Schematic illustration of ferroptosis prevention by tocotrienols and tocopherols. Tocotrienols (T3) and tocopherols (Toc) scavenge lipid hydroperoxyl radicals (LOO•) and lipid alkoxyl radicals (LO•), forming lipid hydroperoxides (LOOH) and lipid alcohols (LOH), respectively. Compared to tocopherols, tocotrienols exhibit superior antioxidant activities, more effectively inhibit lipid peroxidation, and show greater potency in suppressing ferroptosis.

In this study, we demonstrated that, compared with tocopherol, tocotrienol results in superior ferroptosis inhibition, highlighting its potential as a more effective antioxidant for combating ferroptosis (Fig. 6).

Tocotrienols and tocopherols share structural similarities but differ in their biological activities. Tocotrienols have three double bonds in the isoprenoid side chain, which allow them to integrate into cell membranes more readily^23^. The unique ability of tocotrienol to be incorporated into membranes may make it more efficient at stabilizing lipid peroxidation processes and reducing oxidative damage. This characteristic may enhance their ability to scavenge lipid radicals and suppress lipid peroxidation, a critical step in the execution of ferroptosis. Our results demonstrated that tocotrienols effectively prevent ferroptosis and reduce lipid peroxidation, whereas tocopherols have a less pronounced effect. In addition, cytotoxicity of vitamin E analogs, including tocotrienols, is comparable to or lower than that of commonly studied ferroptosis inhibitors, such as Lip-1, Fer-1, and deferoxamine, making them more favorable for therapeutic application.

Apart from the antioxidant capacity, the cellular uptake and distribution of each vitamin E analog may also contribute to its antiferroptotic effects. Our previous study demonstrates that tocotrienols exhibit higher cellular uptake rates than tocopherols^13^, which may partially explain their stronger antiferroptotic effects. However, the previous study also indicated that δ-tocotrienol is taken up by cells approximately eight times more efficiently than α-tocotrienol^13^, yet α-tocotrienol remains the most potent ferroptosis inhibitor among them in the present study. This discrepancy suggests that additional biological mechanisms may contribute to the antiferroptotic effects of vitamin E analogs. For example, differences in the intracellular and organelle localization of each analog after cellular uptake, their distribution within or near cellular membranes, and variations in their intracellular recycling rates might contribute to the observed effects. Further investigation into these factors is warranted in future studies.

Although our study demonstrates that tocotrienols are more effective than tocopherols in inhibiting ferroptosis in vitro, the comparative antiferroptotic effect in vivo remains to be determined. In *vivo*, differences in uptake mechanisms, bioavailability, and tissue distribution among individual vitamin E analogs may influence their impact. For instance, tocopherols, particularly α-tocopherol, are actively taken up via α-tocopherol transfer protein (α-TTP)^31,32^, whereas tocotrienols have a lower affinity for α-TTP, resulting in reduced systemic bioavailability^32^. Recent findings also suggest that a glycosaminoglycan-driven lipoprotein uptake pathway, especially for α-tocopherol, contributes to cellular defense against ferroptosis^33^. However, whether this pathway contributes to the uptake of tocotrienols remains unclear. Additionally, animal studies indicate that tocopherols and tocotrienols exhibit distinct tissue distribution patterns^34^, which may lead to organ-specific differences in their antiferroptotic effects.

In conclusion, our findings provide direct evidence that tocotrienols are more potent inhibitors of ferroptosis than tocopherols. Given their superior ferroptosis-suppressing effects, tocotrienols hold promise as potential therapeutic agents for diseases associated with ferroptosis.

## Methods

### Chemicals and materials

*(1S,3R)*-RSL3 (RSL3: Cayman, Cay19288), erastin (Merck, 329,600), *L-*buthionine sulfoximine (BSO: Sigma Aldrich, B2515), 4-hydroxytamoxifen (4-OH-TAM, Sigma Aldrich, H7904), liproxstatin-1 (Lip-1: Selleckchem, S7699), 6-hydroxy-2,5,7,8-tetramethylchroman-2-carboxylic acid (Trolox: Sigma Aldrich, 238,813), resazurin sodium salt (Sigma Aldrich, R7017), deferoxamine mesylate salt (Sigma Aldrich, 138–14-7), ferrostatin-1 (Fer-1: Sigma Aldrich, SML0583), fetal bovine serum (FBS: Gibco. 10,270–106), penicillin streptomycin (Gibco, 15,140–122), 2, 2’-azobis(2-methylpropionamidine) dihydrochloride (AAPH, Cayman, 82,235), STY-BODIPY (Cayman, 27,089, and BODIPY 581/591 C11 (Thermo, D3861) were purchased. α, β, γ, δ-tocopherol and α, β, γ, δ-tocotrienol were kindly provided by Mitsubishi Chemical Corporation, Ltd. (Tokyo, Japan). Human fibrosarcoma HT-1080 cells were obtained from ATCC (CCL-121). Pfa1 cells are murine embryonic fibroblasts engineered with a tamoxifen-inducible Cre recombinase system that allows conditional deletion of the *Gpx4* gene^29^.

### Cell culture

The cells were maintained in DMEM (high glucose, with GlutaMAX, Gibco, 10,566,024) supplemented with 10% FBS and 1% penicillin/streptomycin, and cultured in a 37 °C incubator with a humidified atmosphere of 5% CO_2_.

### Cell viability assay

Cell viability was assessed using the resazurin reduction assay^26^. After the designated treatment period, the culture medium was removed, and 100 μL of culture medium containing 0.004% resazurin (w/v) was added to each well. The plates were then incubated at 37 °C for 4 h, allowing viable cells to metabolize resazurin into the fluorescent product, resorufin. The fluorescence intensity was measured using a microplate reader (Infinite F200Pro, Tecan) with an excitation wavelength of 535 nm and an emission wavelength of 595 nm. Background fluorescence was measured in blank wells containing resazurin medium without cells. Values lower than the blank were set to zero for subsequent calculations.

### Ferroptosis induction

Ferroptosis was induced in HT-1080 and Pfa1 cells using the ferroptosis inducers RSL3, erastin, or BSO at predetermined concentrations. Cells were seeded into 96-well plates (2,000 cells/well for RSL3 and erastin; 500 cells/well for BSO) and incubated for 24 h to allow attachment. The culture medium was then replaced with fresh medium containing ferroptosis inducers and/or ferroptosis inhibitors (e.g., vitamin E analogs). For co-treatment experiments, ferroptosis inhibitors were added 1 h prior to the addition of ferroptosis inducers. Cells were then incubated for time periods specific to each inducer (24 h for RSL3, 48 h for erastin and 72 h for BSO). For 4-OH-TAM treatment, Pfa1 cells (500 cells/well) were seeded in 4-OH-TAM (1 μM)-containing medium, and cells were incubated for 72 h. Following incubation, cell viability was assessed.

### LDH release assay

LDH release was assessed using the Cytotoxicity Detection kit (Roche, 11,644,793,001). HT-1080 cells (2000 cells/well) were seeded in 96-well plates and incubated overnight. On the following day, the medium was replaced with fresh medium containing ferroptosis inducers (erastin 1 μM or BSO 10 μM) with or without vitamin E analogs, and the cells were incubated for 48 h. After the incubation, 25 μL of culture supernatant from each well was transferred to a new 96-well plate for LDH measurement. An equal volume (25 μL) of LDH reaction solution was added to each well, and the mixture was incubated at room temperature in the dark for 30 min. After incubation, the samples were diluted fourfold with PBS, and absorbance was measured at 510 nm using a microplate reader (Infinite F200Pro, Tecan). The relative LDH release rate (%) was calculated with the value for the group without inhibitor set as 100%.

### Immunoblotting

Cells were lysed in LCW lysis buffer (0.5% Triton X-100, 0.5% sodium deoxycholate salt, 150 mM NaCl, 20 mM Tris–HCl, 10 mM EDTA and 30 mM sodium pyrophosphate tetrabasic decahydrate, pH 7.5) supplemented with a protease and phosphatase inhibitor mixture (cOmplete and PhosSTOP; Roche, 04,693,116,001 and 4,906,837,001). The lysates were incubated on ice for 30 min and centrifuged at 20,000 × g for 30 min at 4 °C. The supernatants were collected, mixed with 6 × SDS sample buffer (375 mM Tris–HCl, pH 6.8, 9% SDS, 50% glycerol, 9% β-mercaptoethanol and 0.03% bromophenol blue) and heated at 55 ˚C for 3 min. Samples were resolved by SDS-PAGE on 12% polyacrylamide gels and transferred onto PVDF membranes (Bio-Rad, 12,023,954) using a semidry transfer system. Membranes were blocked with 5% skim milk (Wako, 190–12,865) in TBS-T (20 mM Tris–HCl, 150 mM NaCl, and 0.1% Tween-20) for 1 h at room temperature and then incubated overnight at 4 °C with primary antibodies against GPX4 (1:1,000; ab125066, Abcam) and valosin-containing protein (VCP, 1:10,000; ab11433, Abcam). After washing with TBS-T, membranes were incubated with HRP-linked secondary antibody (Anti-rabbit IgG, 1:5,000, 7074S, Cell signaling technology,) for 1 h at room temperature. Chemiluminescence imaging was performed using ATTA WSE-6100H LuminoGraph I (ATTO Corporation).

### FENIX assay

The inhibitory effects of tocopherols and tocotrienols on lipid peroxidation were assessed using the fluorescence-enabled inhibited autoxidation (FENIX) assay^25^ with slight modifications. Phosphatidylcholine (Sigma, P7443) was dissolved in chloroform to prepare a 100 mg/mL (equivalent to 129 mM) solution. The solvent was evaporated under an N_2_ stream to form a thin lipid film on the wall of the glass vial. The lipid film was then hydrated with PBS (pH 7.4), and the lipid suspension was subjected to 15 freeze–thaw cycles in liquid nitrogen and a 37ºC water bath. The suspension was subsequently extruded 20 times using a mini-extruder (Avanti Research) fitted with 100 nm polycarbonate membranes (Avanti Research, 610,005-1Ea) to obtain liposomes. A mixture containing a 1 mM liposome suspension, 1 μM STY-BODIPY, and the test compounds was prepared in PBS (pH 7.4). Then, 99 μL aliquots of the mixture were incubated in a black 96-well plate at 37ºC for 10 min using a microplate reader (SpectraMax M5, Molecular Devices). Autooxidation was initiated by adding 1 μL of AAPH to achieve a final concentration of 1 mM. The fluorescence kinetics of the oxidized form of STY-BODIPY were recorded every 5 min with an excitation wavelength of 488 nm and an emission wavelength of 518 nm.

### BODIPY 581/591 C11 assay

The inhibitory effects of tocopherols and tocotrienols on lipid peroxidation in cells were assessed using BODIPY 581/591 C11 assay^35^ with slight modifications. HT-1080 cells (4.0 × 10^4^ cells per well) were seeded on 12-well plates. On the following day, ferroptosis inhibitors were added 1 h prior to treatment with RSL3 (0.5 μM for 2 h). After treatment, the medium was replaced with fresh medium containing BODIPY 581/591 C11 (5 µM), and the cells were incubated for 30 min at 37 °C. Cells were then washed with PBS, trypsinized and resuspended in 500 µL culture medium for flow cytometric analysis using CytoFLEX (Beckman Coulter). Fluorescence data were collected using the FITC channel (488-nm laser) and detected with a 525/40 nm bandpass filter to measure oxidized BODIPY 581/591 C11. A minimum of 10,000 events were recorded for each sample. Relative lipid peroxidation value was calculated based on the mean fluorescence (background-subtracted), with the RSL3(–) group set to 1.0. Data were acquired using CytoExpert (Beckman Coulter) and analyzed with FlowJo v10 (BD Bioscience).

### Evaluation of cellular toxicity

HT-1080 and Pfa1 cells were seeded into 96-well plates at 1,000 cells/well and incubated for 24 h. The next day, the medium was replaced with media containing compounds at concentrations ranging from 0 to 500 μM in a total volume of 100 μL and incubated for 72 h. After incubation, cell viability was assessed.

### Statistics

Statistical information for individual experiments can be found in the corresponding figure legends. Graphs were created using GraphPad Prism v10 (GraphPad Software).

## Supplementary Information

Supplementary Information

## Data Availability

The datasets used and/or analysed during the current study available from the corresponding author on reasonable request.
